# Mesozooplankton Graze on Cyanobacteria in the Amazon River Plume and Western Tropical North Atlantic

**DOI:** 10.3389/fmicb.2017.01436

**Published:** 2017-08-03

**Authors:** Brandon J. Conroy, Deborah K. Steinberg, Bongkuen Song, Andrew Kalmbach, Edward J. Carpenter, Rachel A. Foster

**Affiliations:** ^1^Department of Biological Sciences, Virginia Institute of Marine Science, College of William & Mary, Williamsburg VA, United States; ^2^Department of Biology, Romberg Tiburon Center for Environmental Studies, San Francisco State University, Tiburon CA, United States; ^3^Ocean Sciences, University of California, Santa Cruz, Santa Cruz CA, United States; ^4^Department of Ecology, Environment and Plant Sciences, Stockholm University Stockholm, Sweden

**Keywords:** mesozooplankton, cyanobacteria, diazotroph, grazing, Amazon River plume, North Atlantic Ocean, nitrogen incorporation

## Abstract

Diazotrophic cyanobacteria, those capable of fixing di-nitrogen (N_2_), are considered one of the major sources of new nitrogen (N) in the oligotrophic tropical ocean, but direct incorporation of diazotrophic N into food webs has not been fully examined. In the Amazon River-influenced western tropical North Atlantic (WTNA), diatom diazotroph associations (DDAs) and the filamentous colonial diazotrophs *Trichodesmium* have seasonally high abundances. We sampled epipelagic mesozooplankton in the Amazon River plume and WTNA in May–June 2010 to investigate direct grazing by mesozooplankton on two DDA populations: *Richelia* associated with *Rhizosolenia* diatoms (het-1) and *Hemiaulus* diatoms (het-2), and on *Trichodesmium* using highly specific qPCR assays targeting nitrogenase genes (*nifH*). Both DDAs and *Trichodesmium* occurred in zooplankton gut contents, with higher detection of het-2 predominantly in calanoid copepods (2.33–16.76 *nifH* copies organism^-1^). Abundance of *Trichodesmium* was low (2.21–4.03 *nifH* copies organism^-1^), but they were consistently detected at high salinity stations (>35) in calanoid copepods. This suggests direct grazing on DDAs, *Trichodesmium* filaments and colonies, or consumption as part of sinking aggregates, is common. In parallel with the qPCR approach, a next generation sequencing analysis of 16S rRNA genes identified that cyanobacterial assemblage associated with zooplankton guts was dominated by the non-diazotrophic unicellular phylotypes *Synechococcus* (56%) and *Prochlorococcus* (26%). However, in two separate calanoid copepod samples, two unicellular diazotrophs Candidatus *Atelocyanobacterium thalassa* (UCYN-A) and *Crocosphaera watsonii* (UCYN-B) were present, respectively, as a small component of cyanobacterial assemblages (<2%). This study represents the first evidence of consumption of DDAs, *Trichodesmium*, and unicellular cyanobacteria by calanoid copepods in an area of the WTNA known for high carbon export. These diazotroph populations are quantitatively important in the global N budget, widespread and hence, the next step is to accurately quantify grazing. Nonetheless, these results highlight a direct pathway of diazotrophic N into the food web and have important implications for biogeochemical cycles, particularly oligotrophic regions where N_2_ fixation is the main source of new nitrogen.

## Introduction

Primary production in the marine environment is mostly limited by nitrogen availability (Gruber, 2008). In the open ocean dissolved inorganic nitrogen (DIN) is rare, diazotrophic organisms, those able to utilize N_2_ through the process of biological N_2_ fixation, play a significant role as drivers of primary production by provision of new nitrogen (N) (Dugdale and Goering, 1967). Often the most abundant and best investigated of open ocean diazotrophs are cyanobacteria. The non-heterocyst forming, filamentous cyanobacterium *Trichodesmium* has been a major focus and is especially dominant throughout the tropical and subtropical oceans. Estimations of N_2_ fixation by *Trichodesmium* vary, but globally it is a significant source of new N to the open ocean (Capone et al., 1997; Capone, 2001; Sohm et al., 2011b) through varied pathways including exudation (Glibert and Bronk, 1994; Bronk and Steinberg, 2008), programmed cell death (Berman-Frank et al., 2004) and viral lysis (Hewson et al., 2004). Diatom diazotroph associations (DDAs) are also of considerable interest because they are capable of expansive blooms and high rates of N_2_ fixation (Carpenter et al., 1999; Foster et al., 2007; Subramaniam et al., 2008; Villareal et al., 2012). High densities, including blooms, have been observed in many tropical river plumes including the Amazon (Foster et al., 2007; Subramaniam et al., 2008), Congo (Foster et al., 2009), and Mekong (Grosse et al., 2010; Bombar et al., 2011). Blooms of DDAs are also important for enhancing carbon export from surface waters (Cooley and Yager, 2006; Subramaniam et al., 2008; Karl et al., 2012; Yeung et al., 2012).

More recently other diazotrophs including Archaea, heterotrophic bacteria, and several lineages of unicellular cyanobacteria have been identified as equally important N_2_ fixers in the open ocean (Zehr et al., 1998, 2003; Leigh, 2000; Goebel et al., 2010; Halm et al., 2012; Thompson et al., 2012). Work with the prior two is still limited (Sohm et al., 2011b) but unicellular cyanobacteria have broad distributions, and thus the range of N_2_ fixation now includes areas of the world’s ocean outside the tropical and subtropical latitudes (e.g., cooler temperate regions) (Needoba et al., 2007; Goebel et al., 2010; Moisander et al., 2010; Sohm et al., 2011b; Farnelid et al., 2016). High N_2_-fixation rates have been directly measured (Martínez-Pérez et al., 2016) for unicellular diazotrophs that are comparable to previous estimates for *Trichodesmium* (Falcon et al., 2004; Montoya et al., 2004) indicating an additional source of diazotrophically derived nitrogen available in the food web.

However, less is known about the impacts of N_2_-fixers on secondary production. For example, compared to other dominant primary producers, grazing by zooplankton is not considered a major pathway for new N to enter the food web from *Trichodesmium*. Toxicity and unpalatability of *Trichodesmium* are thought to be the major deterrents for grazing by most zooplankton (O’Neil and Roman, 1994; O’Neil, 1998; Carpenter and Capone, 2008), although a few genera of harpacticoid copepods are known to feed on *Trichodesmium* (O’Neil and Roman, 1994; O’Neil et al., 1996; O’Neil, 1998). Moreover, the harpacticoid, *Macrosetella gracilis* relies on *Trichodesmium*, using the colonies as a habitat and a substrate for juvenile development (Björnberg, 1965; Bottger-Schnack and Schnack, 1989; O’Neil and Roman, 1994; Sheridan et al., 2002). The calanoid copepod *Acartia tonsa* was also observed to graze on *Trichodesmium* during a bloom along the coast of North Carolina (Guo and Tester, 1994). A recent review by Turner (2014) highlights the varied responses of zooplankton to toxic algae (including a number of cyanobacteria) with grazing impact varying considerably according to predator and prey species and their environment.

Low δ^15^N ratios of both suspended particles and zooplankton in tropical and subtropical waters (Montoya et al., 2002; Landrum et al., 2011; Karl et al., 2012; Hunt et al., 2016) indicate diazotrophic nitrogen (N_D_) incorporation into zooplankton and the food web. However, this methodology does not distinguish which zooplankton actively consumes diazotrophs or the source of N_D_ incorporated into the zooplankton (e.g., DDAs, *Trichodesmium*, unicellular cyanobacteria). As mentioned previously *Trichodesmium* is not considered a major prey item for oceanic zooplankton and, to our knowledge, no prior studies have observed direct consumption of DDAs by zooplankton. Furthermore, a modeling study by Stukel et al. (2014) suggests that release from grazing pressure due to decreased zooplankton populations stimulates DDA blooms in the WTNA. A number of studies (e.g., Pfannkuche and Lochte, 1993; Gorsky et al., 1999; Wilson and Steinberg, 2010; Stukel et al., 2013) have found the non-diazotrophic unicellular cyanobacterium, *Synechococcus*, is grazed by zooplankton either individually or as a component of aggregates. Comparatively only two studies show direct consumption of the diazotrophic unicellular groups. In the coastal North Atlantic, Scavotto et al. (2015) showed the symbiotic diazotrophic cyanobacterium *Atelocyanobacterium thalassa* (UCYN-A) occurred in copepod guts and suggested copepod grazers targeted the larger (4–5 μm) prymnesiophyte host rather than individual cells of the cyanobacteria (<1 μm). More recently, Hunt et al. (2016) showed in a mesocosm experiment performed in an oligotrophic lagoon in the southwest Pacific that one of the diazotrophic unicellular cyanobacteria groups (UCYN-C) was grazed by zooplankton and potentially contributed 28–73% N_D_ to the food web. We are not aware of any studies to date indicating grazing on the diazotroph *Crocosphaera watsonii* (UCYN-B), which can be found in singular, aggregate, or symbiotic (with diatoms) forms (Carpenter and Janson, 2000; Foster et al., 2013; Thompson and Zehr, 2013), although UCYN-B does produce extracellular polysaccharide (EPS) which may act as a grazing deterrent (Liu and Buskey, 2000; Sohm et al., 2011a).

In contrast to the paucity of studies on zooplankton-diazotroph grazing in the marine environment, freshwater literature provides many examples of these interactions. The number of toxic cyanobacteria blooms in freshwater and estuarine systems have increased due to eutrophication and climate change (Paerl and Otten, 2013). Subsequently, considerable effort has focused on cyanobacteria bloom successional patterns and fate (Ger et al., 2014; Gerphagnon et al., 2015). Similar to the results of O’Neil and Roman (1994) and O’Neil (1998) in the marine environment, many freshwater studies have suggested morphology, toxicity, and unpalatability of cyanobacteria as deterrents to zooplankton grazing (von Elert et al., 2003; Ger et al., 2014). Yet size-selective herbivorous copepods target cyanobacteria (Bouvy et al., 2001), and Kâ et al. (2011) observed that copepods, cladocerans, and rotifers actively graze and fragment larger filamentous cyanobacteria. While there are differences between freshwater and marine planktonic food webs, it seems improbable that dominant freshwater grazers (e.g., copepods and cladocerans) are able to adapt and consume large-scale, often toxic, cyanobacterial blooms as a food source (Kâ et al., 2011; Gerphagnon et al., 2015) while their marine counterparts entirely avoid feeding on cyanobacteria.

The goal of this study was to investigate if mesozooplankton grazers in the Amazon River plume-influenced WTNA directly grazed upon diazotrophic organisms. In concert with this study, we recently reported elevated mesozooplankton grazing in the Amazon River plume relative to non-plume influenced waters (Conroy et al., 2016), although the community-based pigment approach used did not distinguish the exact prey items. Here, we used a molecular quantification method targeting the *nifH* genes, encoding nitrogenase enzymes, to detect two DDA populations and *Trichodesmium* in the gut contents of zooplankton in the WTNA. We also used a next generation sequencing (NGS) analysis of 16S rRNA genes to investigate the cyanobacterial composition in zooplankton.

## Materials and Methods

### Study Area

**FIGURE 1:**
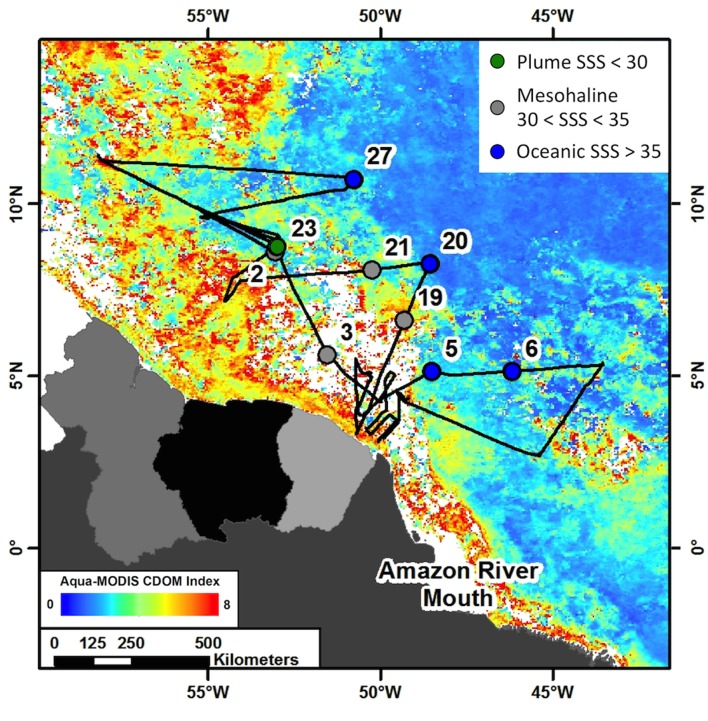
Cruise track from ANACONDAS cruise in May–June, 2010 with stations sampled for analyses used in this study labeled. Cruise track is overlaid on monthly averaged chromophoric dissolved organic matter (CDOM) concentration from Aqua-MODIS satellite data (oceancolor.gsfc.nasa.gov). Colors represent station categories based on sea surface salinity (SSS). Stations with SSS < 30 are considered “plume” and shown in green, stations with 30 < SSS < 35 are considered “mesohaline” and shown in gray and stations with SSS > 35 are considered “oceanic” and shown in blue.

Samples were collected from 9 stations in the Amazon River plume-influenced region of the WTNA (between 0 and 13°N and 44–57°W) as part of the Amazon INfluence on the Atlantic: CarbOn export from Nitrogen fixation by DiAtom Symbioses (ANACONDAS) project. Data presented here are from a cruise aboard the *R/V Knorr* May 22–June 24, 2010, during the period of peak plume discharge (**Figure 1**) and a large scale DDA bloom of *Hemiaulus-Richelia* (Goes et al., 2014). Underway monitoring of sea surface salinity (SSS) and photosynthetic pigments as well as satellite monitoring of plume indicators such as chromophoric dissolved organic matter (CDOM) were the measurements used to determine station location. Station categorizations were determined by SSS at time of the respective tow for each sample. We sampled from 1 plume station (23) with SSS < 30; 4 mesohaline stations (2, 3, 19, and 21) with SSS between 30 and 35; and 4 oceanic stations (5, 6, 20, and 27) with SSS > 35.

### Mesozooplankton Collection

Mesozooplankton were collected with a 1-m Multiple Opening and Closing Net and Environmental Sensing System (MOCNESS; Wiebe et al., 1976) fitted with ten 202 μm mesh nets. Daytime tows were performed between 1000 and 1400 h while nighttime tows were collected between 2200 and 0200 h (local time). Four depth intervals within the top 150 m were sampled with the MOCNESS, although molecular analysis was performed on animals collected from the two shallowest depth intervals (0–25 and 25–50 m) since shipboard observations and cell abundances detected the highest number of microscopically identified diazotrophs (e.g., *Trichodesmium*, DDAs) in the upper 50 m. Once the nets were onboard, zooplankton were immediately anesthetized with carbonated water to prevent gut evacuation (Gannon and Gannon, 1975). Samples were subsequently split into either ¼ or ½ of the total sample volume using a Folsom plankton splitter, then size fractionated using nested sieves into the following size fractions: 0.2–0.5, 0.5–1.0, 1.0–2.0, 2.0–5.0, and >5.0 mm. Each size fraction was then concentrated onto a pre-weighed, 0.2 mm Nitex mesh filter, rinsed with Milli-Q to remove salt, and frozen at -80°C until processed in the laboratory.

### Phytoplankton Cell Abundances

To compare grazing at stations with different diazotroph assemblages, mesozooplankton selection for molecular analysis was based on phytoplankton distribution from Goes et al. (2014) utilizing pigment analysis, physical and chemical properties described in Loick-Wilde et al. (2016), and phytoplankton cell counts presented here. Cells for microscopy counts were collected directly from the CTD at distinct depths (5–6) in the upper 150 m and approximately at midday (local noon). Stations 2, 23, and 27 were sampled once per day for 2 days. The entire contents of the Niskin bottles were gravity filtered onto a 47 mm diameter Poretics (Millipore) membrane filter with a pore size of 10 μm. The filter was removed and mounted onto an oversize microscope slide (75 mm × 50 mm × 1 mm) and examined under 400× magnification using a Zeiss Axioskop Epifluorescence microscope (Zeiss, Berlin, Germany). Colonial and single trichomes of *Trichodesmium*, as well as the symbiotic *Richelia*, were identified by phycoerythrin and chlorophyll a (Chl *a*) excitation with green (510–560 nm) and blue (450–490 nm) excitation wavelengths, respectively. The diatoms associated with *Richelia* were identified as *Hemiaulus* or *Rhizosolenia* spp. based on cell ultrastructure. In many instances the entire filter was scanned and symbiotic diatom cells enumerated as number of *Richelia* heterocysts L^-1^ for each respective diatom and for *Trichodesmium* as colonies L^-1^ (col L^-1^) or single trichomes L^-1^. In some instances when symbiotic cells were at high densities, several smaller grid areas (62.5 μm^2^) of the filter were scanned, and at least 1000 cells were enumerated and corrections to cell abundance were made by the area scanned (Supplementary Table S1).

### DNA Extraction and Quantitative PCR (qPCR) Assays

Zooplankton from the 0.5–1.0 and 1.0–2.0 mm size fractions were selected after visual inspection under a stereomicroscope. Harpacticoid copepods (*Macrosetella gracilis* and *Miracia* spp.) and the decapod shrimp *Lucifer faxoni* were identified to species or genus level, while calanoid copepods, fish larvae, and decapod larvae from the family Thalassinidae were not (Supplementary Table S2). These targets were chosen based on mesozooplankton community composition of each station, onboard microscopic counts from the cruise, pigment concentrations (Goes et al., 2014), and stable isotope analysis (Loick-Wilde et al., 2015) for the same cruise. Calanoid copepods were selected, as they were present across all salinities and with only a few exceptions were the most abundant taxa in all samples, while the harpacticoid copepods were selected because of known associations with *Trichodesmium*. The *L. faxoni*, fish, and decapod larvae were included given their periodic high abundance. The taxa analyzed were mostly calanoid copepods (and proportionately representative of the zooplankton community composition; Conroy and Steinberg, in preparation), with the relative taxonomic representation as follows: calanoid copepods (69.4%), harpacticoid copepods (19.4%), decapod larvae including crab megalopae and Thalassinidae larvae (4.2%), fish larvae (4.2%), and *L. faxoni* (2.8%) (Supplementary Table S2).

Animals were sorted and placed in autoclaved artificial seawater and inspected for exterior contamination with phytodetritus in appendages and mouthparts. Animals were picked clean of any obvious large particles using a needle and forceps, and following the procedure of Boling et al. (2012) subsequently rinsed five times with autoclaved artificial seawater. Animals were inspected again for contaminating phytoplankton and cyanobacteria using blue (450–490 nm) and green (510–560 nm) excitation filters on an epifluorescent compound microscope at 200–450× magnifications. This procedure ensured animals chosen for molecular analysis were phytoplankton-free on their exterior. Between 25 and 50 animals were pooled per DNA extraction (Supplementary Table S1); the number of individuals varied depending on size and availability of target and the results from preliminary PCR assays which determined the lowest number of pooled individuals needed for consistent amplification (see below). Samples were extracted using a modification to the Qiagen DNeasy^®^ Blood and Tissue Kit Animal Tissue (Spin-Column) protocol. Briefly, a 12-h lysis step was performed and all recommended reagent volumes were halved during the extraction. The final elution volume was 35 μl in the provided AE buffer.

We performed qPCR assays for three of the major diazotrophs in the WTNA, two DDAs (het-1, *Richelia* associated with *Rhizosolenia* diatoms and het-2, *Richelia* associated with *Hemiaulus* diatoms), and *Trichodesmium* spp. using the previously described oligonucleotides (Church et al., 2005a,b; Foster et al., 2007) and a modified TaqMAn assay (see below). A total of 72 samples were analyzed for all three targets (het-1, het-2, *Trichodesmium*) with the exception of two calanoid copepod samples collected from St. 2 that were not run with the het-1 assay, and six samples from St. 3 were not run with the *Trichodesmium* due to low template (Supplementary Table S2). In preliminary attempts (data not shown) to optimize the extraction and detection by qPCR, we identified a minimum number of individuals for replicable amplification. From those results we used a minimum of 25 pooled individuals per extraction but, unless limited by abundance of the taxa in a sample, we pooled 50 individuals per extraction (Supplementary Table S2).

For all TaqMan PCR, the 12 μL reactions contained 6.25 μL TaqMan 2X Master Mix (Applied Biosystems), 0.5 μL forward and reverse 0.5 μM primers, 0.25 μL fluorogenic 0.5 μM probe, 2.5 μL of nuclease free water, and 2 μL of DNA template. All reactions were run in triplicate, and for the no-template controls, 2 mL of 5-kD filtered nuclease free water was added to each reaction. All qPCR assays were performed on an ABI 7500 Fast machine (Applied Biosystems) with the following thermal cycling conditions: 50°C for 2 min, 95°C for 10 min, and 45 cycles of 95°C for 15 s, followed by 60°C for 1 min. Gene copy abundances were calculated from the mean cycle threshold (*C*_t_) value of three replicates and the standard curve for the appropriate primer and probe set. For each primer and probe set, triplicate standard curves were made from 10-fold dilution series ranging from 10^8^ to 1 gene copies per reaction. The standard curves were made from linearized plasmids of the target *nifH*. Regression analyses of the results (number of cycles = *C*_t_) of the duplicate standard curves were analyzed in Excel. In some samples only one of the three replicates produced an amplification signal; these were noted as detectable, but not quantifiable (*dnq*). For samples where two or three of the replicates amplified the values were averaged and reported as *nifH*-gene copies per organism. We note that while we report gene copies per organism, as is convention with qPCR, we do not scale our numbers to an estimation of feeding rate. Instead we consider amplification of our targets as confirmation of direct grazing on either DDAs or *Trichodesmium* (see detailed explanation in Discussion).

### 16S rRNA Gene Sequencing of Zooplankton

Sequencing of 16S rRNA genes in zooplankton (containing their gut contents) followed a modified protocol (Arfken et al., 2015) using the Ion Torrent PGM sequencer (Life Technologies). DNA concentrations from the extractions were measured on a NanoDrop 2000 (Thermo Scientific) and PCR was performed on samples normalized to 5 ng μl^-1^ per reaction, except for fish larvae, which were normalized to 20 ng μl^-1^ (see Supplementary Table S3 for samples included in sequencing). The V4 hypervariable region of 16S rRNA genes was targeted with 515F and 805R primers for PCR reactions using GoTaq Green Master Mix (Promega) (Caporaso et al., 2011). The 805R primers have barcodes with fusion sequences while the 515F contains fusion sequences only. PCR reactions were performed as follows: an initial denaturing step for 3 min at 95°C, then 30 cycles of 30 s at 95°C, 1 min at 55°C, 1 min at 72°C, and finally at 72°C for 5 min. PCR products for each sample were then combined and sequencing was conducted. Barcoded samples were sequenced using the Ion Torrent 400 base pair (bp) sequencing protocol with samples pooled onto a 316 chip. We note that for the 16S rRNA analysis we consider our results representative of the zooplankton “microbiome,” similar to other studies (Scavotto et al., 2015; Shoemaker and Moisander, 2015), however, we focus our results on the cyanobacteria and are confident that the cleaning methods adapted from Boling et al. (2012) were adequate enough and our results represent the cyanobacteria consumed by the various mesozooplankton.

### Statistical Analyses and Bioinformatics Pipeline

Sequencing output was downloaded from the Torrent Server using Torrent Suite v3.0 to obtain the FastQ file. A total of 33 libraries were created using the barcoded sequences. Denoising was performed with Acacia (Bragg et al., 2012) and the open-source bioinformatics program Mothur (Schloss et al., 2009) was used to trim and align the sequences. Chimera sequences were removed using UCHIME and remaining sequences for each library were classified using the Greengenes database^1^ at confidence of greater than or equal to 0.80. Relative abundance of each taxa was calculated by dividing the number of classified sequences by total number of sequences in each library. All cyanobacteria sequences identified through Greengenes database were subsampled and subsequently reclassified through the SILVAngs pipeline^2^ with the SILVA 128 reference library (Quast et al., 2013). Additionally, principal coordinate analysis (PCoA) was performed on square-root transformed relative abundance data in PRIMER 7 to identify patterns in microbial diversity between libraries. The raw sequence reads have been submitted to National Center for Biotechnology Information^3^ and are available under Bioproject number PRJNA387277.

## Results

### Plume and Oceanic Diazotroph Phytoplankton Assemblages

At all stations, except Station 6, the *Hemiaulus-Richelia* DDA was more abundant than the *Rhizosolenia-Richelia* DDA. As such, below we only report the former, although all cell counts are available in Supplementary Table S1 for comparison.

**FIGURE 2:**
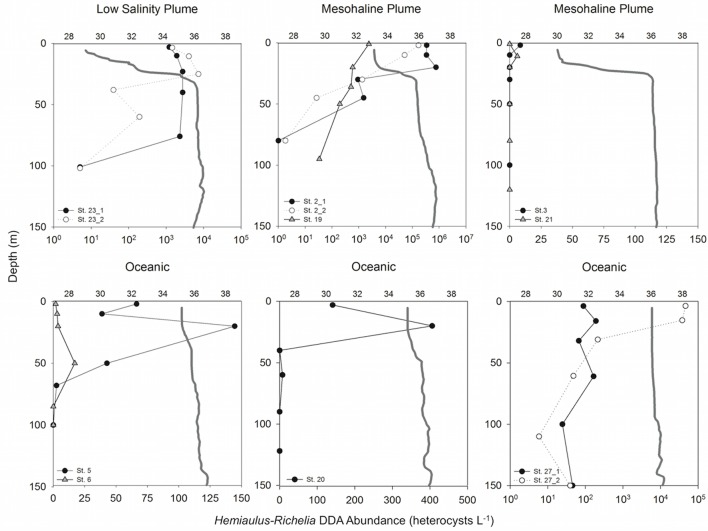
Depth profiles of *Hemiaulus-Richelia* DDA abundance (heterocysts L^-1^) for each station sampled with representative salinity profiles (gray solid lines). For Stations 23, 2, and 27, open circles are abundances for the second day sampled. Upper *x*-axes indicate salinity; note different scale bars on lower *x*-axes for abundance.

The diazotroph assemblage within the low salinity plume (SSS < 30) at station 23 was dominated by *Hemiaulus-Richelia* DDAs (39.4–7.29 × 10^3^ heterocysts L^-1^; Supplementary Table S1) primarily in the upper 75 m (**Figure 2**). At several stations in the mesohaline plume (30 > SSS > 35; stations 2, 3, 19, and 21), cell counts for stations 2 and 19 showed the DDA assemblage described by Goes et al. (2014) was dominated by *Hemiaulus-Richelia*, and the highest cell density of the cruise (7.66 × 10^5^ heterocysts L^-1^; Supplementary Table S1) occurred at 20 m at Station 2 (**Figure 2**). At station 19 in the upper 100 m, *Hemiaulus-Richelia* and *Trichodesmium* were similar in abundance, ranging from 34.0–2.39 × 10^3^ heterocysts L^-1^ and 415–1.59 × 10^3^ trichomes L^-1^, respectively (Supplementary Table S1). In comparison, stations 3 and 21 had low DDA abundances. At station 3, *Hemiaulus-Richelia* abundance (8.4 heterocysts L^-1^) was low in surface waters (∼2 m), free-living *Richelia* cells in the surface 20 m were the highest observed on the cruise (34.9–338 heterocysts L^-1^; Supplementary Table S2). Goes et al. (2014) characterized the phytoplankton community of Station 21 as dominated by *Trichodesmium* and *Synechococcus* spp. based on pigments, and microscopy confirmed *Trichodesmium* as the most dominant cyanobacterial diazotroph in the phytoplankton assemblage, with 4.6–141 trichomes L^-1^ in the surface 50 m (**Figure 2**).

Oceanic stations (SSS > 35; stations 5, 6, 20, and 27) were dominated by *Trichodesmium* and *Synechococcus* spp. *Trichodesmium* was present at all four oceanic stations but was not always the most abundant diazotroph in the phytoplankton assemblage (Supplementary Table S1) as previously described in Goes et al. (2014) based on pigments. At Stations 6 and 27 *Hemiaulus-Richelia* were more abundant than *Trichodesmium*, particularly at Station 27 with a 1–2 order of magnitude difference (5.9–4.66 × 10^4^ heterocysts L^-1^ vs. 25.6–1.25 × 10^3^ trichomes L^-1^ in the surface 150 m, respectively; Supplementary Table S1).

### Quantitative PCR of Gut Contents

*DDAs*– Both the het-1 (*Richelia* associated with *Rhizosolenia* diatoms) and het-2 (*Richelia* associated with *Hemiaulus* diatoms), targets were successfully amplified from the gut content extractions (**Table 1** and Supplementary Table S2). Het-2 was the most common target amplified across all extractions, with a total of 25 samples amplified–24 from calanoid copepods and one from *Macrosetella gracilis*. Of those, 11 (10 calanoids and 1 *M. gracilis*), were detectable not quantifiable (*dnq*). The remaining samples detected het-2 with a range of 1.6–16.8 *nifH*-copies/organism. All stations except for station 3, the furthest inshore mesohaline station, had samples that amplified the het-2 target.

**Table 1:** Percentage of samples (n), for each taxa, exhibiting positive qPCR amplification of Het-1, Het-2, and *Trichodesmium* spp. in molecular assays.

Taxa	*n*	Het-1 (%)	Het-2 (%)	*Trichodesmium* spp. (%)
Calanoid copepods	50	4.0	48.0	28.0
Harpacticoids copepods	14	0.0	7.1	28.6
Decapod larvae	3	0.0	0.0	66.7
Fish larvae	3	0.0	0.0	0.0
*L. faxoni*	2	0.0	0.0	0.0

*Decapod larvae include both crab megalopae and Thalassinidae.*

In comparison to het-2, the other DDA, het-1, was detected only in calanoid copepods collected at night at Station 19 (**Table 1** and Supplementary Table S2). Each of the two size fractions at this station had very low detection with the smaller calanoid copepods *dnq* and larger calanoid copepods 0.10 *nifH* copies/organism. No pattern was observed between night (*n* = 12) and day (*n* = 13) samples for het-2, whereas the only het-1 detection occurred at night (Supplementary Table S2).

*Trichodesmium*– The *nifH* of *Trichodesmium* spp. was also successfully detected in the gut contents of the mesozooplankton samples (**Table 1** and Supplementary Table S2). Twenty samples out of 72 (14%) showed amplification: 14 in calanoid copepods (7 *dnq*), 4 in *M. gracilis* (3 *dnq*), and 2 in crab megalopae (1 *dnq*) (**Figure 2**). All oceanic stations (St. 5, 6, 20, and 2; **Figure 1**), as well as one mesohaline station (St. 19), included in this analysis detected *Trichodesmium*. Values ranged from 1.1 to 4.0 *Trichodesmium nifH* copies/organism with the highest value found in crab megalopae. No pattern was observed between day (*n* = 11) and night (*n* = 9) samples for detecting *Trichodesmium*.

### Characterization of Zooplankton Gut Contents

Sample selection for 16S rRNA NGS analysis was guided by our qPCR assays and resulted in the 33 samples selected for analysis. Not all samples with amplification from the qPCR were included due to template limitation, but a representative number of samples provided over 1.7 million sequences for analysis (see Supplementary Table S3 for NGS samples).

**FIGURE 3:**
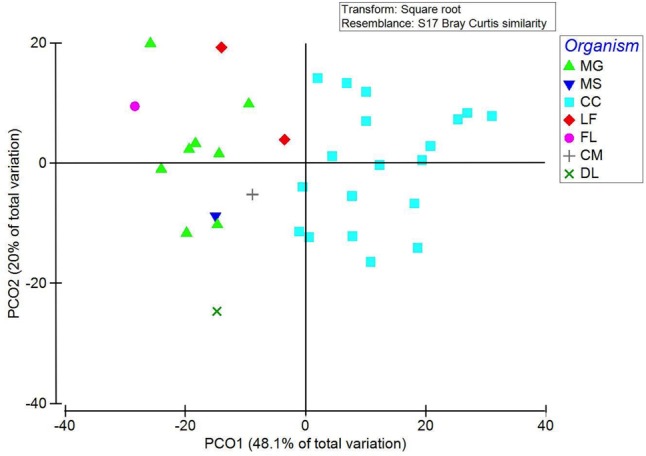
PCoA of 16S rRNA NGS of gut microbial community shown by zooplankton taxonomic group (organism). PCO1 and PCO2 explain 48.1 and 20% of the variation, respectively. Samples primarily grouped by taxonomy with the major clusters being calanoid copepods (blue squares) and harpacticoid copepods including both *Macrosetella gracilis* and *Miracia* spp. (green triangles). Zooplankton taxonomic groups are abbreviated as follows: MG, *Macrosetella gracilis*; MS, *Miracia* spp.; CC, calanoid copepod; LF, *Lucifer faxoni*; FL, fish larvae; CM, crab megalopae, and DL, decapod larvae.

PCoA of 16S rRNA sequences showed a tight clustering of two groups (**Figure 3**). Calanoid copepods (blue squares in **Figure 3**) and all harpacticoid copepods (green triangles in **Figure 3**) clustered by taxa. Other variables analyzed (salinity, size fraction, depth interval, time of day) resulted in no significant grouping.

**FIGURE 4:**
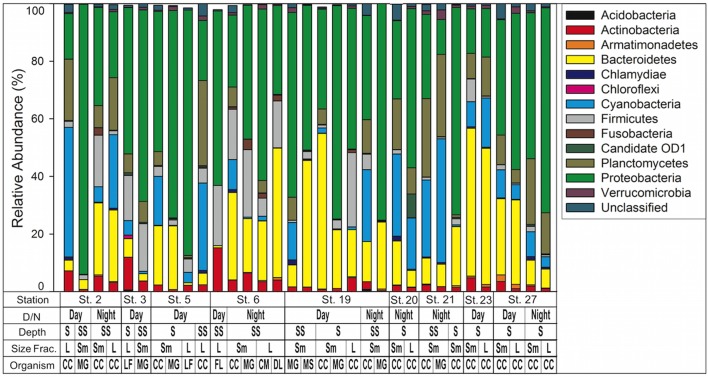
Bacterial community composition by phylum as determined by 16S rRNA NGS analysis. Phyla are listed in key to right. Key below graph identifies each sample with categories as follows: D/N, day or night; Depth S = Surface 0–25 m, SS = Sub Surface 25–50 m; Size fraction *L* = 1.0–2.0 mm, *S*m = 0.5–1.0 mm; Organism CC, calanoid copepod; MG, *Macrosetella gracilis*; MS, *Miracia* spp; LF, *Lucifer faxoni*; FL, fish larvae; CM, crab megalopae, and DL, decapod larvae.

Proteobacteria were the most abundant phyla represented in our samples, with 16 of the 33 samples having over 50% of sequences associated with proteobacteria (**Figure 4**). Cyanobacteria represented between 0 and 45% of bacterial composition across all samples and represented 11.3% of total sequences (Supplementary Table S3). In the non-calanoid copepod samples (*n* = 14), the decapod shrimp *L. faxoni*, had cyanobacterial sequences associated with its gut which represented >1% of total sequences. Harpactacoid copepods *M. gracilis* and *Miracia* spp. had cyanobacterial sequences present but were all <1% of total sequences, and the fish larvae sample analyzed was devoid of cyanobacterial sequences (**Figure 4** and Supplementary Table S3).

**FIGURE 5:**
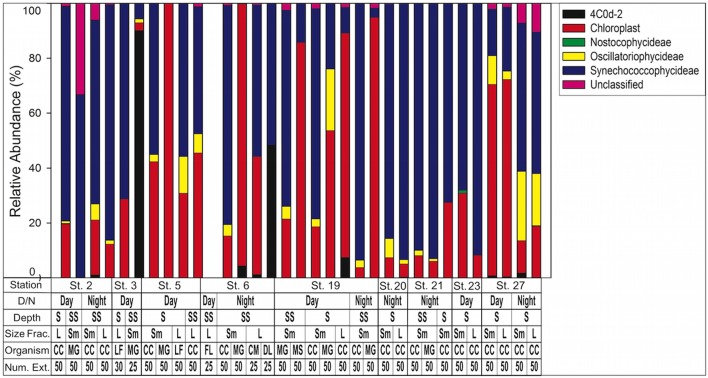
Cyanobacteria assemblage composition determined by 16S rRNA NGS analysis. Classes are listed in key to right. Note chloroplasts are included in the Green Genes database although not technically cyanobacteria. 4C0d-2 are closely related to cyanobacteria but recently proposed candidates for the new phylum melainabacteria. Key below graph identifies each sample with categories as follows: D/N, day or night; Depth S = Surface 0–25 m, SS = Sub Surface 25–50 m; Size fraction *L* = 1.0–2.0 mm, *S*m = 0.5–1.0 mm; Organism CC, calanoid copepod; MG, *Macrosetella gracilis*; MS, *Miracia* spp.; LF, *Lucifer faxoni*; FL, Fish Larvae; DL, Decapod Larvae; CM, Crab Megalopae; Num Ext., animals per extraction.

When composition of only cyanobacterial sequences (*n* = 197,298) was analyzed with the GreenGenes database, the most abundant class was Synechococcophycideae (**Figure 5**). When investigated to genus level, the most abundant within this class was *Synechococcus* (*n* = 83,745, representing 42.4% of all cyanobacterial sequences). *Prochlorococcus* was the next largest contributor at the genus level (*n* = 22,734 representing 11.4% of all cyanobacterial sequences) although 21.2% of cyanobacterial sequences in Synechococcophycideae were unidentifiable to genus classification. Additionally, in a several samples chloroplast sequences composed up to 100% of the cyanobacterial sequences (**Figure 5**).

Reclassification of cyanobacterial sequences based on SILVA 128 database agreed that *Synechococcus* and *Prochlorococcus* were still the most abundant representatives (56 and 26%, respectively). However, at station 5 and station 21 UCYN-A and UCYN-B sequences were found within the smallest size fraction calanoid copepod samples, representing 1–2% of sequences.

## Discussion

Studying the pathway of diazotrophically derived production (N_D_) in the marine food web is challenging; however, with several of the molecular-based approaches, new insights can be achieved. For example, using *nifH* qPCR assays with highly specific oligonucleotides allowed detection of DDAs and *Trichodesmium* in the gut contents of mesozooplankton. Moreover, our parallel 16S rRNA NGS libraries further elucidated the greater cyanobacterial diversity in zooplankton guts. Templates derived from the harpacticoid copepod *M. gracilis*, commonly considered the major grazer of *Trichodesmium*, were often below detection for the *Trichodesmium* qPCR assays. Although our sample size for harpacticoid copepods was low. We also found evidence that crab megalopae consume *Trichodesmium*. Finally, our 16S rRNA NGS analysis concluded that mesozooplankton taxa frequently consume the non-diazotrophic picoplanktonic cyanobacteria *Synechococcus* and *Prochlorococcus* spp., and are capable of consumption of other diazotrophic unicellular cyanobacteria such as UCYN-A and UCYN-B. However, these results provide insights on cyanobacteria-zooplankton interactions with important implications for the pathways of N_D_ entering marine food webs and for N cycling in the WTNA.

### Diazotroph Consumption by Mesozooplankton

*DDAs*–Stable isotope studies in the subtropical and tropical Atlantic Ocean (Montoya et al., 2002; Landrum et al., 2011; Loick-Wilde et al., 2015), including the region of our study, established that new N attributed to N_2_-fixation from diazotrophs is incorporated in the planktonic food web, but the pathways were not elucidated (i.e., through the microbial loop, exudation, or grazing). The qPCR results of the gut content show N_D_ can enter the food web via consumption of DDAs, as both DDA targets (het-1 and het-2) were detected in the zooplankton gut contents. This is the first evidence for consumption of DDAs. These results are consistent with DDA distributions observed in our microscopy, where het-2 was the most dominant of the two DDAs present during our study, as well as in a prior studies of the Amazon-influenced WTNA (Carpenter et al., 1999; Foster et al., 2007; Luo et al., 2012). Consumption of DDAs is also consistent with the decreased δ^15^N content of zooplankton reported by Loick-Wilde et al. (2016) in the mesohaline plume, particularly at Stations 2 and 19 (their Figure 9). At St. 19, the only station where het-1 was detected in the guts of mesozooplankton, microscope counts observed both *Hemiaulus* and *Rhizosolenia* symbioses were present. However, *Hemiaulus-Richelia* symbioses (het-2) were 1–2 orders of magnitude more abundant and found deeper in the water column than the *Rhizosolenia-Richelia* symbiosis (het-1).

The pattern of DDA distribution in the WTNA largely follows the nutrient and surface salinity gradients outlined in Subramaniam et al. (2008), with DDAs occurring in the mesohaline region of the plume (Goes et al., 2014; Loick-Wilde et al., 2015). However, the diazotrophic cyanobacterial counts and molecular results reported here indicate there is not a strict boundary between the salinity regimes for DDA presence, particularly along the transition from mesohaline to oceanic waters. All four of the oceanic stations included in this study had low abundances of het-2 symbioses along with amplification of this target in zooplankton gut contents. These stations were characterized as an oceanic assemblage with abundant *Trichodesmium* and *Synechococcus* by Goes et al. (2014) (note Station 6 was not included in their study). Therefore, het-2 consumption at all four oceanic stations sampled is unexpected and can be explained by plume meanders or development of frontal zones between mesohaline and oceanic waters. These physical features are common in river plumes and represent areas of zooplankton aggregation and increased grazing (Woodson et al., 2005; True et al., 2015; Woodson and Litvin, 2015) and could promote episodic, opportunistic feeding in oceanic mesozooplankton.

Our results, while novel, are not unexpected. Conroy et al. (2016) characterized the mesozooplankton food web from grazing rate estimations at low salinity plume and mesohaline waters with SSS < 33 as an export style food web. Export food webs are characterized by shorter diatom and mesozooplankton food chains compared to retention food webs dominated by microzooplankton and the microbial loop (Michaels and Silver, 1988; Legendre and Michaud, 1998; Pomeroy et al., 2007). Both DDAs investigated form chains, often suggested as a grazing deterrent, yet copepods actively graze diatoms of similar size (Bergkvist et al., 2012). Therefore in the plume and mesohaline stations DDA consumption supports a shorter export food web; DDA consumption we observed at oceanic stations is likely more occasional, but when it occurs could increase export.

*Trichodesmium* were consumed by zooplankton at all four oceanic stations, as well as at two mesohaline stations. Calanoid copepods were again the primary consumers, although the highest *nifH* gene copies organism^-1^ were amplified from crab megalopae. Despite the higher abundance of *M. gracilis* at oceanic stations relative to plume stations (Conroy et al., in preparation) and its known association with, and grazing on, *Trichodesmium* (O’Neil and Roman, 1994; O’Neil et al., 1996; O’Neil, 1998), this species only showed one amplification for *Trichodesmium* that was not *dnq* (**Figure 2** and Supplementary Table S2). This is not to say *M. gracilis* does not graze on *Trichodesmium*, which was reported to be a pathway of atmospheric nitrogen incorporation into zooplankton in the eastern tropical Atlantic (Sandel et al., 2015), but that this result is an artifact of our methodology, discussed in detail below.

Prior to our study there was little evidence of calanoid copepod grazing on *Trichodesmium*, and a general consensus emerged that it did not occur or was severely limited (Capone et al., 1997; Carpenter and Capone, 2008). The detection of *Trichodesmium* in the guts of calanoid copepods in the WTNA is novel and builds on other limited evidence for calanoid grazing on *Trichodesmium* globally (Hawser et al., 1992; Guo and Tester, 1994). Calanoid copepods were typically the most abundant organism across all stations in the three smallest size fractions (0.2–0.5, 0.5–1.0, and 1.0–2.0 mm) (Conroy et al., in preparation), as also found in zooplankton community composition studies in the subtropical North Atlantic (Steinberg et al., 2008; Eden et al., 2009). Thus, grazing by copepods on *Trichodesmium* in the oligotrophic tropical and subtropical ocean where *Trichodesmium* is abundant (Capone et al., 1997, 2005) has potential for a significant input of N_D_ into the food web. Furthermore, these results support the observed low δ^15^N of mesozooplankton at oceanic stations (Loick-Wilde et al., 2015), was due, at least in part, to direct grazing on *Trichodesmium*. Similar evidence was recently reported in the Eastern tropical North Atlantic where δ^15^N of zooplankton was correlated with *Trichodesmium* spp. colony abundance (Sandel et al., 2015).

Other work from stable isotope analysis suggests that decreases in δ^15^N in *Trichodesmium*-dominated waters is due to N_D_ exudation and incorporation into the food web via the microbial loop (Montoya et al., 2002; Mulholland, 2007) rather than direct grazing, predominately due their potentially allelopathic toxins (Hawser et al., 1992). In the eastern tropical North Atlantic Ocean, Sandel et al. (2015) observed decreased δ^15^N in carnivorous, omnivorous, and *Trichodesmium-*grazing copepods. Aside from the known *Trichodesmium* grazers included in that study (*M. gracilis* and *M. efferata*) they did not suggest direct consumption of *Trichodesmium* by other copepod species. However, Hawser et al. (1992) showed that toxicity was species dependent and that not all *Trichodesmium* spp. are toxic to zooplankton. Furthermore, Guo and Tester (1994) investigated the effect of *Trichodesmium* sp. on the calanoid copepod *Acartia tonsa* after a meander from the Gulf Stream transported *Trichodesmium* inshore toward Albemarle Sound, North Carolina. They found no toxic effects of *Trichodesmium* on *A. tonsa* when fed healthy cells, but observed toxic effects, including distended guts and mortality, when fed aging or senescing cells or when treated with a filtered cell homogenate (Guo and Tester, 1994). Given the ephemeral exposure *A. tonsa* has to *Trichodesmium* as a predominantly coastal copepod, it is reasonable that calanoid copepods exposed to *Trichodesmium* during development in the open ocean would have a similar ability to consume *Trichodesmium*. Furthermore, a study from the tropical North Atlantic between the Cape Verde Islands and Barbados reports that δ^15^N values for zooplankton and *Trichodesmium* are similar, so that direct consumption is the likeliest explanation (McClelland et al., 2003, see their Figure 6). Zooplankton grazing on *Trichodesmium* is further supported by zooplankton-cyanobacteria interactions from freshwater habitats. Kâ et al. (2011) performed feeding experiments with calanoid copepods, cladocerans, and rotifers and found grazing could be a significant factor in controlling filamentous cyanobacteria. While we are unable to scale our numbers to estimate the grazing impact on *Trichodesmium* (see below), our results support that *Trichodesmium* is directly consumed by mesozooplankton.

### Microbial and Cyanobacterial Diversity Associated with Zooplankton

Pairing the highly specific qPCR approach with a 16S rRNA NGS approach provided insight into the broader zooplankton-cyanobacterial dynamics in the WTNA. Our 16S rRNA sequences show abundant phyla that varied between samples, with proteobacteria and cyanobacteria consistently a large percentage of all sample sequences. Similar to findings of Shoemaker and Moisander (2015) from the subtropical North Atlantic, our results indicate a distinct partitioning of microbes based on taxonomic groups rather than environmental factors (**Figure 3**). While an understanding of the complete microbiome of zooplankton is important (Scavotto et al., 2015; Shoemaker and Moisander, 2015), particularly in the oligotrophic ocean where microenvironments (e.g., zooplankton) support unique bacterial assemblages (Azam and Malfatti, 2007), we limit the scope of our discussion to the cyanobacteria given their potential role in primary production, N_2_ fixation, and carbon export.

Predominance of *Synechococcus* and to a lesser extent *Prochlorococcus* sequences within the most abundant cyanobacteria class (**Figure 5**) is similar to results from the same cruise investigating diversity of free-living and particle-associated cells. Metatranscriptomic studies conducted by Satinsky et al. (2014) showed that the prokaryotic assemblage was dominated by the Synechococcaceae family of cyanobacteria (Satinsky et al., 2014, See Supplementary Figure S1 of that study). Those results are limited to one station, which we did not sample; however, it does provide insight into why our sequences were dominated by *Synechococcus* and *Prochlorococcus. Synechococcus* and *Prochlorococcus* are globally abundant (Flombaum et al., 2013) but generally considered too small to be directly consumed by most mesozooplankton. On the other hand, consumption of the latter are often considered indirectly through feeding on marine snow aggregates or fecal pellets (Wilson and Steinberg, 2010), or by ingesting microzooplankton which previously consumed small cells. Satinsky et al. (2014) showed a higher percentage of Synechococcaceae cyanobacteria in “particle-associated” sequences compared to “free-living,” while HPLC pigment data showed abundant *Synechococcus* throughout the plume and WTNA (Goes et al., 2014). We conclude that predominance of *Synechococcus* and *Prochlorococcus* sequences in the crustacean zooplankton are likely from consumption of cyanobacteria-containing aggregates and/or consumption of microzooplankton, which had consumed picocyanobacteria previously.

The presence of UCYN-A and UCYN-B sequences in two different calanoid copepod samples indicates a pathway of N_D_ directly into the food web (Zehr, 2011; Thompson et al., 2012, 2014). Scavotto et al. (2015) is the only other study to show consumption of UCYN-A by zooplankton with their results showing the calanoid copepod *Acartia* spp. regularly had UCYN-A in full gut contents. To our knowledge there are no studies reporting UCYN-B consumption by zooplankton. UCYN-A is comparable in size to *Synechococcus* and *Prochlorococcus*, but UCYN-A is suggested to be an obligate, or at least mutualistic, symbiont with a larger haptophyte (Thompson et al., 2012; Farnelid et al., 2016) and UCYN-B can be found in free-living, colonial and symbioses with diatoms. Therefore, UCYN-A and UCYN-B may be directly targeted by zooplankton or consumed as components of larger sinking aggregates in the water column.

We did not analyze water column UCYN-A or UCYN-B abundance; however, results from prior studies in the tropical Atlantic suggest both are widely distributed, particularly in the eastern Atlantic and outer most regions of the plume-influenced WTNA (Foster et al., 2007; Goebel et al., 2010; Moisander et al., 2010). Globally, UCYN-A and UCYN-B are observed throughout the tropics and thus our results suggest a broad potential pathway for N_D_ from unicellular diazotrophs into planktonic food webs, particularly in areas such as the south Pacific where high concentrations for both are observed (Moisander et al., 2010; Farnelid et al., 2016).

Lastly, we offer explanations for the chloroplast sequences identified in the cyanobacterial sequences (**Figure 5**). Our NGS protocol required a PCR amplification step and it is possible that these chloroplasts could represent a bias introduced during amplification (Acinas et al., 2005). More likely is that the sequences represent phytoplankton, likely cyanobacteria, that were ingested but are not included in the most recent GreenGenes reference taxonomy (McDonald et al., 2012).

### Perspectives for Scaling up to Grazing and Method Improvements

While molecular methods to quantify zooplankton grazing rates are becoming more common (Nejstgaard et al., 2008; Troedsson et al., 2009; Cleary et al., 2012, 2016; Shoemaker and Moisander, 2015), we hesitate to extend our qPCR results beyond a qualitative assessment of grazing. The *nifH* Taqman probes are highly specific, thus other non-targeted organisms consumed, which our NGS analysis confirms, would not be detected by the qPCR assays but could interfere with the amplification (Kanagawa, 2003; Nejstgaard et al., 2008). On the other hand, given the evidence of polyploidy in heterocystous cyanobacteria (Griese et al., 2011; Sukenik et al., 2012) and recently observed in *Trichodesmium* (Sargent et al., 2016) there is potential to overestimate the *nifH* copy number by qPCR. However, target DNA degradation and inefficient DNA extraction in the gut is a significant factor in underestimating zooplankton grazing, particularly in copepods, with molecular methods (Simonelli et al., 2009; Troedsson et al., 2009). Conroy et al. (2016) observed elevated grazing in both the low salinity plume stations and in the intermediate mesohaline stations utilizing the gut pigment method, yet our qPCR assays all yielded low gene copies per organism regardless of station category. Similarly, Nejstgaard et al. (2008) found a pattern of underrepresentation of gut contents when comparing qPCR estimates to gut pigment estimates. Regardless of the low gene copies, we are confident our results are indicative of grazing on DDAs and *Trichodesmium* given the high specificity of our qPCR assays and our sanitation techniques. In order to account for low gene copies due to gut degradation, we suggest future work include controlled grazing experiments using a culture of the targeted diazotroph for analyses so that a differential length amplification qPCR (dla-qPCR) method, similar to that utilized in Troedsson et al. (2009), could account for DNA degradation, and be used to estimate grazing rate on diazotrophs. Furthermore, the molecular methods utilized in this study could be paired with fluorescent *in situ* hybridization as well as analysis of gut contents through traditional electron microscopy. Lastly, there is the potential for contamination from environmental DNA (eDNA) that at present is not accounted for in this or other studies. While likely a low percentage of the genetic material analyzed, methodologies such as the one used here, that will likely have low amplification, need to consider a method for determining contamination from eDNA.

## Conclusion

Diazotrophic nitrogen incorporation into the planktonic food web has long been observed through the use of nitrogen stable isotope analysis. While an extremely robust method, stable isotope analysis lacks the nuance to determine the exact pathways N_D_ enters the planktonic food web. We provide direct evidence that two DDAs, *Hemiaulus-Richelia* and *Rhizosolenia-Richelia*, are consumed by mesozooplankton. We further show that *Trichodesmium* is consumed by calanoid and harpacticoid copepods, as well as some decapod larvae. Lastly, we show that unicellular cyanobacteria, particularly non-diazotrophic *Synechococcus* and *Prochlorococcus*, as well as diazotrophic UCYN-A and UCYN-B, are consumed by zooplankton, likely as components of aggregates or in symbioses with microalgae (e.g., UCYN-A). Grazing on UCYN-A and UCYN-B provides an additional and previously undocumented pathway for N_D_ incorporation into the food web in the WTNA. This study has important implications for our understanding of cyanobacterial-zooplankton dynamics in a changing ocean. Increased stratification, due to warming surface waters, is expected to elevate the importance of N_2_-fixation in the oligotrophic open ocean (Doney et al., 2012), and our results suggest that mesozooplankton that consume diazotrophs would likely benefit. Our results suggest the need for investigation beyond the WTNA, as in other areas with DDA blooms such as the Congo, Niger, and Mekong River plumes (Foster et al., 2009; Grosse et al., 2010; Bombar et al., 2011), the South Pacific Ocean (Turk-Kubo et al., 2015), as well as globally where *Trichodesmium* (Capone et al., 1997, 2005) and unicellular cyanobacteria are important diazotrophs. Questions do remain, however, concerning what other mesozooplankton taxa target diazotrophs, and what are their grazing rates and impacts on the bloom-forming DDAs and *Trichodesmium*. Further work in these areas is needed to extend our results to multiple taxa and other regions, and to quantify specific pathways of diazotrophic nitrogen incorporation into the food web.

## Author Contributions

BC, DS, BS, and RF conceived the zooplankton sampling and experimental design. BC, DS, BS, AK, EC, and RF acquired and analyzed the data. BC, DS, and RF wrote the paper, and BC, DS, BS, AK, EC, and RF edited the manuscript. All authors approved the final submitted manuscript.

## Conflict of Interest Statement

The authors declare that the research was conducted in the absence of any commercial or financial relationships that could be construed as a potential conflict of interest.

## Footnotes

1 http://greengenes.lbl.gov/

2 https://www.arb-silva.de/ngs

3 https://www.ncbi.nlm.nih.gov/
