# Supplementary material for: Mesozooplankton Graze on Cyanobacteria in the Amazon River Plume and Western Tropical North Atlantic
Source: Front Microbiol. 2017 Aug 3;8:1436. doi: 10.3389/fmicb.2017.01436 (PMC5540951; doi:10.3389/fmicb.2017.01436)
Supplement: Supplementary file 1 [file Table_1.docx]

Table S1. Depth profiles of *Trichodesmium* (trichomes L^-1^) DDAs, and free living *Richelia* (heterocysts L^-1^) abundances for stations included in molecular analysis.

| Station | Depth (m) | *Trichodesmium* | *Rhizosolenia-Richelia* symbioses | | *Hemiaulus-Richelia* symbioses | Free Living *Richelia* | | |
| --- | --- | --- | --- | --- | --- | --- | --- | --- |
| 2 | 2.0 | 10.1 | 0.0 | 3.44 x 10^5^ | | | 0.0 |  |
| 2 | 10.0 | 137 | 16.9 | 3.39 x 10^5^ | | | 0.0 |  |
| 2 | 20.0 | 455 | 0.0 | 7.66 x 10^5^ | | | 0.0 |  |
| 2 | 30.0 | 55.8 | 0.0 | 935 | | | 0.0 |  |
| 2 | 45.0 | 19.1 | 0.0 | 1.54 x 10^3^ | | | 0.0 |  |
| 2 | 80.0 | 0.0 | 0.0 | 0.0 | | | 0.0 |  |
| 2 | 2.0 | 167 | 0.0 | 1.71 x 10^5^ | | | 0.0 |  |
| 2 | 10.0 | 13.6 | 7.9 | 5.18 x 10^4^ | | | 0.0 |  |
| 2 | 30.0 | 73.5 | 5.7 | 1.36 x 10^3^ | | | 0.0 |  |
| 2 | 45.0 | 16.7 | 1.3 | 26.8 | | | 0.0 |  |
| 2 | 80.0 | 11.9 | 14.7 | 1.8 | | | 0.0 |  |
| 3 | 2.0 | 0.0 | 0.0 | 8.4 | | | 34.9 |  |
| 3 | 10.0 | 27.6 | 0.0 | 0.0 | | | 338 |  |
| 3 | 20.0 | 1.6 | 0.0 | 0.0 | | | 178 |  |
| 3 | 30.0 | 0.0 | 0.0 | 0.0 | | | 0.0 |  |
| 3 | 50.0 | 0.0 | 0.0 | 0.0 | | | 0.0 |  |
| 3 | 100 | 0.0 | 0.0 | 0.0 | | | 0.0 |  |
| 5 | 2.0 | 2.8 | 29.4 | 66.4 | | | 7.6 |  |
| 5 | 10.0 | 5.1 | 18.6 | 38.8 | | | 16.0 |  |
| 5 | 20.0 | 2.5 | 34.3 | 145 | | | 3.8 |  |
| 5 | 50.0 | 5.7 | 19.9 | 42.7 | | | 5.7 |  |
| 5 | 68.0 | 11.4 | 27.9 | 2.5 | | | 6.4 |  |
| 5 | 100 | 7.6 | 0.0 | 0.0 | | | 0.0 |  |
| 6 | 2.0 | 167 | 14.5 | 1.9 | | | 0.0 |  |
| Station | Depth (m) | *Trichodesmium* | *Rhizosolenia-Richelia* symbioses | | *Hemiaulus-Richelia* symbioses | Free Living *Richelia* | | |
| 6 | 10.0 | 44.0 | 21.2 | | 3.0 | 0.0 | | |
| 6 | 20.0 | 204 | 17.1 | | 3.8 | 1.9 | | |
| 6 | 50.0 | 85.6 | 14.2 | | 17.1 | 5.7 | | |
| 6 | 85.0 | 0.0 | 0.0 | | 0.0 | 0.0 | | |
| 6 | 100 | 0.0 | 0.0 | | 0.0 | 0.0 | | |
| 19 | 1.0 | 1.08 x 10^3^ | 45.4 | | 2.39 x 10^3^ | 0.0 | | |
| 19 | 20.0 | 731 | 7.6 | | 595 | 0.0 | | |
| 19 | 36.0 | 1.59 x 10^3^ | 0.0 | | 515 | 0.0 | | |
| 19 | 50.0 | 1.03 x 10^3^ | 0.0 | | 197 | 0.0 | | |
| 19 | 95.0 | 415 | 0.0 | | 34.0 | 0.0 | | |
| 19 | 200 | 0.0 | 0.0 | | 0.0 | 0.0 | | |
| 20 | 3.0 | 19.0 | 8.5 | | 141 | 0.0 | | |
| 20 | 20.0 | 252 | 25.6 | | 406 | 0.0 | | |
| 20 | 40.0 | 68.5 | 7.6 | | 0.0 | 0.0 | | |
| 20 | 60.0 | 60.6 | 0.0 | | 7.6 | 0.0 | | |
| 20 | 90.0 | 1.3 | 0.0 | | 0.0 | 0.0 | | |
| 20 | 122 | 0.0 | 0.0 | | 0.0 | 0.0 | | |
| 21 | 1.0 | 142 | 0.0 | | 0.0 | 0.0 | | |
| 21 | 11.0 | 138 | 0.0 | | 6.1 | 0.0 | | |
| 21 | 20.0 | 86.4 | 3.0 | | 0.0 | 0.0 | | |
| 21 | 50.0 | 4.6 | 0.0 | | 0.0 | 0.0 | | |
| 21 | 80.0 | 0.0 | 0.0 | | 0.0 | 0.0 | | |
| 21 | 120 | 0.0 | 0.0 | | 0.0 | 0.0 | | |
| 23 | 3.0 | 117 | 0.0 | | 1.22 x 10^3^ | 0.0 | | |
| 23 | 10.0 | 3.8 | 0.0 | | 1.92 x 10^3^ | 0.0 | | |
| 23 | 23.0 | 116 | 0.0 | | 2.76 x 10^3^ | 0.0 | | |
| Station | Depth (m) | *Trichodesmium* | *Rhizosolenia-Richelia* symbioses | | *Hemiaulus-Richelia* symbioses | Free Living *Richelia* | | |
| 23 | 40.0 | 1.3 | 1.3 | | 2.76 x 10^3^ | 0.0 | | |
| 23 | 76.0 | 0.0 | 0.0 | | 2.33 x 10^3^ | 0.0 | | |
| 23 | 101 | 10.2 | 0.0 | | 5.1 | 0.0 | | |
| 23 | 3.5 | 0.0 | 7.6 | | 1.46 x 10^3^ | 0.0 | | |
| 23 | 10.5 | 0.0 | 0.0 | | 4.04 x 10^3^ | 0.0 | | |
| 23 | 25.0 | 0.0 | 0.0 | | 7.29 x 10^3^ | 0.0 | | |
| 23 | 38.0 | 0.4 | 0.0 | | 39.4 | 0.0 | | |
| 23 | 60.0 | 151.7 | 0.0 | | 195 | 0.0 | | |
| 23 | 102 | 1.3 | 0.0 | | 5.1 | 0.0 | | |
| 27 | 3.7 | 130.7 | 27.7 | | 88.9 | 0.0 | | |
| 27 | 15.9 | 49.3 | 15.2 | | 192 | 0.0 | | |
| 27 | 32.0 | 628.1 | 36.0 | | 67.2 | 0.0 | | |
| 27 | 61.0 | 91.4 | 5.1 | | 166 | 0.0 | | |
| 27 | 100 | 33.9 | 0.0 | | 24.6 | 0.0 | | |
| 27 | 150 | 2.5 | 0.0 | | 45.7 | 0.0 | | |
| 27 | 3.6 | 1253.7 | 3.8 | | 4.66 x 10^4^ | 0.0 | | |
| 27 | 15.3 | 601.6 | 26.6 | | 3.81 x 10^4^ | 0.0 | | |
| 27 | 31.0 | 768.6 | 0.0 | | 215 | 0.0 | | |
| 27 | 60.6 | 25.6 | 0.0 | | 48.4 | 0.0 | | |
| 27 | 110 | 99.0 | 0.0 | | 5.9 | 0.0 | | |
| 27 | 150 | 85.3 | 23.7 | | 39.6 | 0.0 | | |
