# Supplementary material for: Mesozooplankton Graze on Cyanobacteria in the Amazon River Plume and Western Tropical North Atlantic
Source: Front Microbiol. 2017 Aug 3;8:1436. doi: 10.3389/fmicb.2017.01436 (PMC5540951; doi:10.3389/fmicb.2017.01436)
Supplement: Supplementary file 2 [file Table_2.docx]

Table S2. List of samples utilized in qPCR assays targeting het-1 (*Rhizosolenia- Richelia* DDA) and het-2 (*Hemiaulus-Richelia* DDA) and *Trichodesmium* spp., and estimated number of *nifH* gene copies/organism. Samples are identified by station, coordinates, collection time, size fraction, organism and number of individuals pooled in each DNA extraction. Samples which resulted in detection of only 1 of 3 replicates are indicated as dnq = detected not quantifiable. Samples with 2 of 3 or 3 of 3 replicates are reported as *nifH* gene copies per organism for the respective targets. Numbers in parentheses indicate replicates out of 3 reported with 3 of 3 reported in bold. bd indicates below detection or samples which had no amplification detected. *Macrosetella gracilis* and *Miracia spp.* are harpactacoid copepods, the decapod larvae were members of the family Thalassinidae. Not Run indicates samples which were not assayed for a particular target.

| St. | Lat. | Long. | | Time (local) | Depth Interval (m) | Size Fraction (mm) | Organism | Number Extracted | het-1 *nifH* copies/organism | het-2 *nifH* copies/organism | *Trichodesmium nifH* copies/organism |
| --- | --- | --- | --- | --- | --- | --- | --- | --- | --- | --- | --- |
| 2 | 10.29 | | -54.52 | 11:50 | 0-25 | 0.5-1.0 | Calanoid Copepods | 50 | bd | 2.91 (2) | bd |
| 2 | 10.29 | | -54.52 | 11:50 | 0-25 | 0.5-1.0 | *Macrosetella gracilis* | 25 | bd | bd | bd |
| 2 | 10.29 | | -54.52 | 11:50 | 0-25 | 1.0-2.0 | Calanoid Copepods | 50 | bd | dnq (1) | bd |
| 2 | 10.29 | | -54.52 | 11:50 | 25-50 | 0.5-1.0 | Calanoid Copepods | 50 | bd | bd | bd |
| 2 | 10.29 | | -54.52 | 11:50 | 25-50 | 0.5-1.0 | *Macrosetella gracilis* | 50 | bd | bd | bd |
| 2 | 10.29 | | -54.52 | 11:50 | 25-50 | 1.0-2.0 | Calanoid Copepods | 50 | bd | bd | bd |
|  |  | |  |  |  |  |  |  |  |  |  |
| 2 | 10.30 | | -54.50 | 1:04 | 25-50 | 0.5-1.0 | Calanoid Copepods | 50 | Not Run | bd | bd |
| 2 | 10.30 | | -54.50 | 1:04 | 25-50 | 1.0-2.0 | Calanoid Copepods | 50 | Not Run | bd | dnq (1) |
|  |  | |  |  |  |  |  |  |  |  |  |
| 3 | 7.30 | | -52.99 | 13:55 | 0-25 | 0.5-1.0 | Calanoid Copepods | 50 | bd | bd | Not Run |
| 3 | 7.30 | | -52.99 | 13:55 | 0-25 | 1.0-2.0 | Calanoid Copepods | 20 | bd | bd | Not Run |
| 3 | 7.30 | | -52.99 | 13:55 | 0-25 | 1.0-2.0 | *Lucifer faxoni* | 30 | bd | bd | Not Run |
| 3 | 7.30 | | -52.99 | 13:55 | 25-50 | 0.5-1.0 | Calanoid Copepods | 50 | bd | bd | Not Run |
| 3 | 7.30 | | -52.99 | 13:55 | 25-50 | 0.5-1.0 | *Macrosetella gracilis* | 25 | bd | bd | Not Run |
| 3 | 7.30 | | -52.99 | 13:55 | 25-50 | 1.0-2.0 | Calanoid Copepods | 50 | bd | bd | Not Run |
|  |  | |  |  |  |  |  |  |  |  |  |
| 5 | 6.82 | | -49.98 | 14:09 | 0-25 | 0.5-1.0 | Calanoid Copepods | 50 | bd | **11.57** | dnq (1) |
| 5 | 6.82 | | -49.98 | 14:09 | 0-25 | 0.5-1.0 | Macrosetella gracilis | 50 | bd | dnq (1) | bd |
| 5 | 6.82 | | -49.98 | 14:09 | 0-25 | 1.0-2.0 | Calanoid Copepods | 25 | bd | 2.11 (2) | bd |
| 5 | 6.82 | | -49.98 | 14:09 | 0-25 | 1.0-2.0 | *Lucifer faxoni* | 50 | bd | bd | bd |
| 5 | 6.82 | | -49.98 | 14:09 | 25-50 | 0.5-1.0 | Calanoid Copepods | 50 | bd | **5.87 (3)** | bd |
| 5 | 6.82 | | -49.98 | 14:09 | 25-50 | 0.5-1.0 | Macrosetella gracilis | 50 | bd | bd | dnq (1) |
| 5 | 6.82 | | -49.98 | 14:09 | 25-50 | 1.0-2.0 | Calanoid Copepods | 50 | bd | **3.31 (3)** | bd |
|  |  | |  |  |  |  |  |  |  |  |  |
| 6 | 6.83 | | -47.61 | 15:02 | 0-25 | 0.5-1.0 | Calanoid Copepods | 50 | bd | dnq (1) | dnq (1) |
| 6 | 6.83 | | -47.61 | 15:02 | 0-25 | 0.5-1.0 | Macrosetella gracilis | 50 | bd | bd | dnq (1) |
| 6 | 6.83 | | -47.61 | 15:02 | 0-25 | 1.0-2.0 | Calanoid Copepods | 50 | bd | bd | bd |
| 6 | 6.83 | | -47.61 | 15:02 | 25-50 | 0.5-1.0 | Calanoid Copepods | 50 | bd | 1.61 (2) | bd |
| 6 | 6.83 | | -47.61 | 15:02 | 25-50 | 1.0-2.0 | Calanoid Copepods | 50 | bd | bd | bd |
| 6 | 6.83 | | -47.61 | 15:02 | 25-50 | 1.0-2.0 | Crab Megalopae | 25 | bd | bd | **4.03 (3)** |
| 6 | 6.83 | | -47.61 | 15:02 | 25-50 | 1.0-2.0 | Fish Larvae | 25 | bd | bd | bd |
|  |  | |  |  |  |  |  |  |  |  |  |
| 6 | 6.82 | | -47.62 | 2:09 | 0-25 | 0.5-1.0 | Calanoid Copepods | 50 | bd | bd | bd |
| 6 | 6.82 | | -47.62 | 2:09 | 0-25 | 0.5-1.0 | Macrosetella gracilis | 50 | bd | bd | 1.88 (2) |
| 6 | 6.82 | | -47.62 | 2:09 | 0-25 | 1.0-2.0 | Calanoid Copepods | 50 | bd | bd | bd |
| 6 | 6.82 | | -47.62 | 2:09 | 25-50 | 0.5-1.0 | Calanoid Copepods | 50 | bd | dnq (1) | bd |
| 6 | 6.82 | | -47.62 | 2:09 | 25-50 | 0.5-1.0 | Macrosetella gracilis | 50 | bd | bd | bd |
| 6 | 6.82 | | -47.62 | 2:09 | 25-50 | 1.0-2.0 | Calanoid Copepods | 50 | bd | bd | bd |
| 6 | 6.82 | | -47.62 | 2:09 | 25-50 | 1.0-2.0 | Crab Megalopae | 25 | bd | bd | dnq (1) |
| 6 | 6.82 | | -47.62 | 2:09 | 25-50 | 1.0-2.0 | Thalassinidae | 25 | bd | bd | bd |
|  |  | |  |  |  |  |  |  |  |  |  |
| 19 | 8.29 | | -50.75 | 13:17 | 0-25 | 0.5-1.0 | Calanoid Copepods | 50 | bd | bd | dnq (1) |
| 19 | 8.29 | | -50.75 | 13:17 | 0-25 | 0.5-1.0 | Macrosetella gracilis | 50 | bd | bd | bd |
| 19 | 8.29 | | -50.75 | 13:17 | 0-25 | 0.5-1.0 | Miracia | 50 | bd | bd | bd |
| 19 | 8.29 | | -50.75 | 13:17 | 0-25 | 1.0-2.0 | Calanoid Copepods | 50 | bd | bd | bd |
| 19 | 8.29 | | -50.75 | 13:17 | 25-50 | 0.5-1.0 | Calanoid Copepods | 50 | bd | 3.23 | dnq (2) |
| 19 | 8.29 | | -50.75 | 13:17 | 25-50 | 0.5-1.0 | Macrosetella gracilis | 50 | bd | bd | bd |
| 19 | 8.29 | | -50.75 | 13:17 | 25-50 | 1.0-2.0 | Calanoid Copepods | 50 | bd | bd | bd |
|  |  | |  |  |  |  |  |  |  |  |  |
| 19 | 8.30 | | -50.76 | 1:36 | 0-25 | 0.5-1.0 | Calanoid Copepods | 50 | dnq (1) | 11.03 | dnq (2) |
| 19 | 8.30 | | -50.76 | 1:36 | 0-25 | 1.0-2.0 | Calanoid Copepods | 50 | dnq (2) | 16.76 | dnq (1) |
| 19 | 8.30 | | -50.76 | 1:36 | 0-25 | 1.0-2.0 | Fish Larvae | 25 | bd | bd | bd |
| 19 | 8.30 | | -50.76 | 1:36 | 25-50 | 0.5-1.0 | Calanoid Copepods | 50 | bd | 12.9 | bd |
| 19 | 8.30 | | -50.76 | 1:36 | 25-50 | 0.5-1.0 | Macrosetella gracilis | 50 | bd | bd | bd |
| 19 | 8.30 | | -50.76 | 1:36 | 25-50 | 1.0-2.0 | Calanoid Copepods | 50 | bd | dnq (1) | dnq (2) |
| 19 | 8.30 | | -50.76 | 1:36 | 25-50 | 1.0-2.0 | Fish Larvae | 25 | bd | bd | bd |
|  |  | |  |  |  |  |  |  |  |  |  |
| 20 | 9.98 | | -50.01 | 23:19 | 0-25 | 0.5-1.0 | Calanoid Copepods | 50 | bd | 8.42 | dnq (1) |
| 20 | 9.98 | | -50.01 | 23:19 | 0-25 | 1.0-2.0 | Calanoid Copepods | 50 | bd | bd | bd |
| 20 | 9.98 | | -50.01 | 23:19 | 25-50 | 0.5-1.0 | Calanoid Copepods | 50 | bd | dnq (1) | bd |
| 20 | 9.98 | | -50.01 | 23:19 | 25-50 | 1.0-2.0 | Calanoid Copepods | 50 | bd | dnq (1) | bd |
|  |  | |  |  |  |  |  |  |  |  |  |
| 21 | 9.76 | | -51.70 | 2:21 | 0-25 | 0.5-1.0 | Calanoid Copepods | 50 | bd | dnq (1) | bd |
| 21 | 9.76 | | -51.70 | 2:21 | 0-25 | 1.0-2.0 | Calanoid Copepods | 50 | bd | bd | bd |
| 21 | 9.76 | | -51.70 | 2:21 | 25-50 | 0.5-1.0 | Calanoid Copepods | 50 | bd | dnq (2) | bd |
| 21 | 9.76 | | -51.70 | 2:21 | 25-50 | 0.5-1.0 | Macrosetella gracilis | 50 | bd | bd | bd |
| 21 | 9.76 | | -51.70 | 2:21 | 25-50 | 1.0-2.0 | Calanoid Copepods | 50 | bd | bd | bd |
|  |  | |  |  |  |  |  |  |  |  |  |
| 23 | 10.65 | | -54.40 | 13:09 | 0-25 | 0.5-1.0 | Calanoid Copepods | 50 | bd | dnq (1) | bd |
| 23 | 10.65 | | -54.40 | 13:09 | 0-25 | 1.0-2.0 | Calanoid Copepods | 50 | bd | dnq (2) | bd |
| 23 | 10.65 | | -54.40 | 13:09 | 25-50 | 0.5-1.0 | Calanoid Copepods | 50 | bd | bd | bd |
| 23 | 10.65 | | -54.40 | 13:09 | 25-50 | 1.0-2.0 | Calanoid Copepods | 50 | bd | bd | bd |
|  |  | |  |  |  |  |  |  |  |  |  |
| 27 | 12.43 | | -52.23 | 13:58 | 0-25 | 0.5-1.0 | Calanoid Copepods | 50 | bd | 2.33 | dnq (2) |
| 27 | 12.43 | | -52.23 | 13:58 | 0-25 | 0.5-1.0 | Macrosetella gracilis | 50 | bd | bd | dnq (1) |
| 27 | 12.43 | | -52.23 | 13:58 | 0-25 | 1.0-2.0 | Calanoid Copepods | 50 | bd | bd | 2.21 |
| 27 | 12.43 | | -52.23 | 13:58 | 25-50 | 0.5-1.0 | Calanoid Copepods | 50 | bd | bd | bd |
| 27 | 12.43 | | -52.23 | 13:58 | 25-50 | 1.0-2.0 | Calanoid Copepods | 50 | bd | dnq (1) | dnq (2) |
|  |  | |  |  |  |  |  |  |  |  |  |
| 27 | 12.47 | | -52.24 | 2:05 | 25-50 | 0.5-1.0 | Calanoid Copepods | 50 | bd | bd | bd |
| 27 | 12.47 | | -52.24 | 2:05 | 25-50 | 1.0-2.0 | Calanoid Copepods | 50 | bd | bd | bd |
| 27 | 12.47 | | -52.24 | 2:05 | 25-50 | 0.5-1.0 | Calanoid Copepods | 50 | bd | bd | dnq (1) |
| 27 | 12.47 | | -52.24 | 2:05 | 25-50 | 1.0-2.0 | Calanoid Copepods | 50 | bd | dnq (1) | dnq (2) |
