# Supplementary material for: Mesozooplankton Graze on Cyanobacteria in the Amazon River Plume and Western Tropical North Atlantic
Source: Front Microbiol. 2017 Aug 3;8:1436. doi: 10.3389/fmicb.2017.01436 (PMC5540951; doi:10.3389/fmicb.2017.01436)
Supplement: Supplementary file 3 [file Table_3.docx]

Table S3. Summary of samples and results from the 16S NGS analysis. Sample collection information is reported, including station, the time of day (day or night), depth interval of collection (m) and size fraction (mm), the total number of sequences, cyanobacteria sequences, and the percentage of total sequences represented by cyanobacteria for respective samples.

| Station | Day/Night | Depth Interval (m) | Size Fraction (mm) | Organism | Total Sequences | Cyanobacteria Sequences | Percent Cyanobacteria |
| --- | --- | --- | --- | --- | --- | --- | --- |
| 2 | Day | 0-25 | 1.0-2.0 | Calanoid Copepods | 27,987 | 12,585 | 46.0 |
| 2 | Day | 25-50 | 0.5-1.0 | *Macrosetella gracilis* | 14,747 | 3 | 0.02 |
| 2 | Night | 25-50 | 0.5-1.0 | Calanoid Copepods | 75,024 | 4,005 | 5.34 |
| 2 | Night | 25-50 | 1.0-2.0 | Calanoid Copepods | 111,459 | 28,506 | 25.6 |
| 3 | Day | 0-25 | 1.0-2.0 | *Lucifer faxoni* | 4,204 | 212 | 5.04 |
| 3 | Day | 25-50 | 0.5-1.0 | *Macrosetella gracilis* | 8,665 | 71 | 0.82 |
| 5 | Day | 0-25 | 0.5-1.0 | Calanoid Copepods | 45,771 | 7,775 | 17.0 |
| 5 | Day | 0-25 | 0.5-1.0 | *Macrosetella gracilis* | 37,313 | 64 | 0.17 |
| 5 | Day | 0-25 | 1.0-2.0 | *Lucifer faxoni* | 6,026 | 217 | 3.60 |
| 5 | Day | 25-50 | 1.0-2.0 | Calanoid Copepods | 42,326 | 12,844 | 30.4 |
| 6 | Day | 25-50 | 1.0-2.0 | Fish Larvae | 3,287 | 0 | 0.00 |
| 6 | Night | 25-50 | 0.5-1.0 | Calanoid Copepods | 24,709 | 2,577 | 10.4 |
| 6 | Night | 25-50 | 0.5-1.0 | *Macrosetella gracilis* | 115,046 | 582 | 0.51 |
| 6 | Night | 25-50 | 1.0-2.0 | Crab Megalopae | 82,420 | 1,259 | 1.53 |
| 6 | Night | 25-50 | 1.0-2.0 | Decapod Larvae | 41,497 | 33 | 0.08 |
| 19 | Day | 0-25 | 0.5-1.0 | Calanoid Copepods | 16,601 | 2,152 | 13.0 |
| 19 | Day | 0-25 | 0.5-1.0 | *Macrosetella gracilis* | 52,389 | 155 | 0.30 |
| 19 | Day | 0-25 | 1.0-2.0 | Calanoid Copepods | 54,674 | 999 | 1.83 |
| 19 | Day | 25-50 | 0.5-1.0 | *Macrosetella gracilis* | 33,822 | 67 | 0.20 |
| 19 | Day | 25-50 | 0.5-1.0 | *Miracia sp.* | 40,999 | 371 | 0.90 |
| 19 | Night | 25-50 | 0.5-1.0 | Calanoid Copepods | 7,259 | 1,808 | 24.9 |
| 19 | Night | 25-50 | 0.5-1.0 | *Macrosetella gracilis* | 35,323 | 59 | 0.17 |
| 20 | Night | 0-25 | 0.5-1.0 | Calanoid Copepods | 34,193 | 9,771 | 28.6 |
| 20 | Night | 0-25 | 1.0-2.0 | Calanoid Copepods | 91,767 | 16,297 | 17.8 |
| 21 | Night | 0-25 | 0.5-1.0 | Calanoid Copepods | 69,605 | 18,572 | 26.7 |
| 21 | Night | 25-50 | 0.5-1.0 | Calanoid Copepods | 69,076 | 29,706 | 43.0 |
| 21 | Night | 25-50 | 0.5-1.0 | *Macrosetella gracilis* | 46,608 | 254 | 0.54 |
| 23 | Day | 0-25 | 0.5-1.0 | Calanoid Copepods | 70,097 | 6,100 | 8.70 |
| 23 | Day | 0-25 | 1.0-2.0 | Calanoid Copepods | 86,336 | 14,753 | 17.1 |
| 27 | Day | 0-25 | 0.5-1.0 | Calanoid Copepods | 93,353 | 9,075 | 9.72 |
| 27 | Day | 0-25 | 1.0-2.0 | Calanoid Copepods | 126,192 | 6,437 | 5.10 |
| 27 | Night | 0-25 | 0.5-1.0 | Calanoid Copepods | 67,591 | 5,856 | 8.66 |
| 27 | Night | 0-25 | 1.0-2.0 | Calanoid Copepods | 114,001 | 4,133 | 3.63 |
|  |  |  |  | Total | 1,750,367 | 197,298 | 11.3 |
